# Cross-cultural measurement invariance of the Quality of Life Enjoyment and Satisfaction Questionnaire-Short form across ten countries: the application of Bayesian approximate measurement invariance

**DOI:** 10.1186/s40359-022-00864-y

**Published:** 2022-06-24

**Authors:** Zahra Bagheri, Parisa Chamanpara, Peyman Jafari, Yatan Pal Singh Balhara, Sidharth Arya, Ramdas Ransing, Ana Đorić, Rajna Knez, Tuong-Vi Vu Thi, Truong Ngoc Huong, Helin Yilmaz Kafali, Gamze Erzin, Zahir Vally, Mita Rani Roy Chowdhury, Pawan Sharma, Rabi Shakya, Luís Antônio Monteiro Campos, Anna Rebeka Szczegielniak, Dejan Stevanović

**Affiliations:** 1 Department of Biostatistics, School of Medicine, Shiraz University of Medical Sciences, Shiraz, Iran; 2 Behavioral Addictions Clinic, Department of Psychiatry and National Drug Dependence Treatment Center, All India Institute of Medical Sciences (AIIMS), New Delhi, India; 3 Institute of Mental Health, Pt. Bhagwat Dayal Sharma University of Health Sciences, Rohtak, India; 4 Department of Psychiatry, B K L Walawalkar Rural Medical College, Kasarwadi, Maharashtra India; 5 Department of Psychology, Faculty of Humanities and Social Sciences, Center for Applied Psychology, University of Rijeka, Rijeka, Croatia; 6 Department of Women´S and Children´S Health, Skaraborgs Hospital, Skövde, Sweden; 7 Gillberg Neuropsychiatry Centre, Institute of Neuroscience and Physiology, Sahlgrenska Academy, University of Gothenburg, Gothenburg, Sweden; 8 South Vietnam HIV Addiction Technical Transfer Centre, University of Medicine and Pharmacy, Ho Chi Minh City, Vietnam; 9 Faculty of Public Health, University of Medicine and Pharmacy at Ho Chi Minh City, Ho Chi Minh City, Vietnam; 10 Department of Child and Adolescent Psychiatry, Ankara City Hospital, Ankara, Turkey; 11 Diskapi Training and Research Hospital, Ankara, Turkey; 12 Department of Psychiatry and Neuropsychology, School for Mental Health and Neuroscience, Maastricht University Medical Center, Maastricht, The Netherlands; 13 Department of Clinical Psychology, United Arab Emirates University, Al Ain, United Arab Emirates; 14 United Nations Department of Safety and Security, Cox’s bazar, Bangladesh; 15 Department of Psychiatry, School of Medicine, Patan Academy of Health Sciences, Lalitpur, Nepal; 16 Catholic University of Petrópolis (UCP) and Pontifical Catholic University of Rio de Janeiro, Rio de Janeiro, Brazil; 17 Department of Psychiatric Rehabilitation, Department of Psychiatry and Psychotherapy, Medical University of Silesia, Katowice, Poland; 18 Clinic for Neurology and Psychiatry for Children and Youth, Belgrade, Serbia

**Keywords:** Cross-cultural, Measurement invariance, Quality of life, Psychiatry, Mental health

## Abstract

**Background:**

The Quality of Life Enjoyment and Satisfaction Questionnaire-Short Form (Q-LES-Q-SF) is the most frequently used generic quality of life (QOL) measure in many countries and cultures worldwide. However, no single study has been carried out to investigate whether this questionnaire performs similarly across diverse cultures/countries. Accordingly, this study aimed to assess the cross-cultural measurement invariance of the Q-LES-Q-SF across ten different countries.

**Methods:**

The Q-LES-Q-SF was administrated to a sample of 2822 university students from ten countries: Bangladesh, Brazil, Croatia, India, Nepal, Poland, Serbia, Turkey, the United Arab Emirates, and Vietnam. The Bayesian approximate measurement invariance approach was used to assess the measurement invariance of the Q-LES-Q-SF.

**Results:**

Approximate measurement invariance did not hold across the countries for the Q-LES-Q-SF, with only two out of 14 items being non-invariant; namely items related to doing household and leisure time activities.

**Conclusions:**

Our findings did not support the cross-cultural measurement invariance of the Q-LES-Q-SF; thus, considerable caution is warranted when comparing QOL scores across different countries with this measure. Item rewording and adaptation along with calibrating non-invariant items may narrow these differences and help researchers to create an invariant questionnaire for reliable and valid QOL comparisons across different countries.

## Background

In recent years, quality of life (QOL) has received considerable attention in both clinical and research settings [1]. However, there is little consensus on the definition of QOL due to the complexity of its concept, with more than 100 questionnaires developed over the past decades [1, 2]. Nevertheless, the World Health Organization (WHO) provided a broad definition of QOL which is centered on individual’s subjective perception of the quality of his or her life: "an individual's perception of their position in life in the context of the culture and value systems in which they live and in relation to their goals, expectations, standards and concerns" [3]. According to this definition, QOL is a multidimensional concept in which physical and emotional well-being in addition to social relations, activity of daily living, and overall life satisfaction are conceptualized [1, 2]. Previous studies showed that different values, traditions, and beliefs in different cultures along with environmental conditions and availability of opportunities strongly affect the QOL construct [4, 5]. Accordingly, QOL questionnaires may be sensitive to the language, dialect, customs, beliefs and traditions of local cultures where they are constructed [4, 5].

Translation and more importantly cultural adaptation are indispensable prerequisites for administering a given questionnaire developed in one cultural group to individuals in another culture [4]. One of the key aspects of cultural adaptation of a questionnaire when aiming to conduct cross-cultural research is assessing the assumption of measurement invariance which is known as cross-cultural measurement invariance [6]. Measurement invariance means that individuals from different groups perceived the meaning of items in a QOL questionnaire in the same way, given the same level of underlying QOL [7]. When measurement invariance does not hold, differences in means and other estimates observed across different cultures cannot be relied upon. Because it is not clear whether the observed difference is a true difference in the underlying construct of interest or it is an artificial effect of different implicit or explicit interpretation of items by individuals in different cultures and countries [7]. Moreover, a lack of measurement invariance of a questionnaire can question its validity due to the fact that a fundamental assumption in measurement is that individuals' response to the items of the questionnaire should not be affected by their characteristics such as country, language or culture which are unrelated to the construct being measured [7].

The Quality of Life Enjoyment and Satisfaction Questionnaire-Short Form (Q-LES-Q-SF) is one of the most frequently used generic QOL questionnaires in psychiatric and mental health research and clinical settings, which was developed from the original long form representing the QOL concept widely [8, 9]. A key feature of this questionnaire is that it emphasizes the subjective perceptions of individuals physical, psychological, and social domains of daily life [10]. Furthermore, this questionnaire has been translated in several languages and validated in diverse groups of individuals with different socio-economic backgrounds and lifestyles, as well as with other health conditions besides the psychiatric ones [2, 8–17]. The results of the previous studies revealed that the Q-LES-Q-SF questionnaire exhibited sound psychometric properties and it has been used as a QOL questionnaire in more than 100 peer-reviewed publications [17]. In addition, the measurement invariance of this questionnaire was confirmed across different age, sex, educational level and type of substance dependence in a French sample [12]. However, to the best of our knowledge, no single study has investigated the cross-cultural measurement invariance of this questionnaire. Due to the increasing interest in international QOL research, there is, therefore, a need to investigate the measurement invariance of this questionnaire across various cultures. This study aimed to assess the cross-cultural measurement invariance of the Q-LES-Q-SF questionnaire across ten countries including, Bangladesh, Brazil, Croatia, India, Nepal, Poland, Serbia, Turkey, the United Arab Emirates, and Vietnam, using Bayesian approximate measurement invariance approach.

## Methods

### Participants

Data for the present study were used from an international project which evaluated psychological well-being and Internet use aspects among university students, including QOL collected with the Q-LES-Q-SF [18, 19]. In that project, data was collected via an online survey. It was considered a convenient sampling of students pursuing various graduation courses in colleges/universities across the available countries from rural and urban communities based on the location of the researchers of the project [18, 19]. The same recruitment procedure was followed across all locations. After obtaining the approval from the respective institution from where the students were contacted, the researchers or project’s staff were responsible to advertise and to send a link of the survey to the students [18, 19]. Students were solicited to participate in the survey directly during classes or via students’ organizations. The completion of the survey was voluntary and anonymous [18, 19]. In total, 2822 college/university students completed the Q-LES-Q-SF questionnaire in the following countries: Bangladesh (183), Brazil (127), Croatia (464), India (487), Nepal (165), Poland (161), Serbia (321), Turkey (251), the United Arab Emirates (210), and Vietnam (453). There is no certain rule for sample size calculation in Bayesian approximate measurement invariance approach; however, previous research showed that this method was also appropriate for studies with small sample size (e.g., n = 150) [20]. Accordingly, except for the Brazil the sample size in the other countries seems to be sufficient.

The survey was available in Croatian, English Polish, Portuguese, Spanish, Serbian, Turkish, and Vietnamese. Students participating form Bangladesh, India, Nepal, and the UAE completed the English version of the survey, since the English language is used completely or in part during their educational courses in the respective colleges/university and there was no need for additional language versions. The only exclusion criterion for the study was not providing informed consent. The study was approved by an institutional board/ethical committee relevant to the authors’ institutions. A signed informed consent form was obtained from all participants prior to starting the data collection. For all details on the sampling see Balhara et al., 2019 [18] and Stevanovic et al., 2020 [19].

### Questionnaire

#### Q-LES-Q-SF

The Q-LES-Q-SF is a 16-item, self-report questionnaire that evaluates QOL and satisfaction in several domains. The first 14 items assess satisfaction with (1) physical health, (2) mood, (3) work, (4) household activities, (5) social relationships, (6) family relationships, (7) leisure activities, (8) daily functioning, (9) sexual life, (10) economic status, (11) living/housing situation, (12) ability to get around physically, (13) vision, and (14) overall well-being. The last two items measure medications, and overall satisfaction and contentment. The items are scored on a 5-point Likert scale from 1 (not at all or never) to 5 (frequently or all the time), with higher scores indicating better enjoyment and satisfaction with life. The total score of the questionnaire is the sum of these 14 items ranging from 14 to 70 and expressed as a percentage based on the maximum total score of the items completed (0–100). The last two items, which are not included in the total score, are about medications and overall life satisfaction and were added to the short form for clinical reasons [8, 11]. Accordingly, we assessed the measurement invariance of the first 14 items in our study.

## Statistical analysis

One-way ANOVA and Chi-square statistics were applied to investigate whether participants from different countries differed significantly in terms of their age and gender, respectively. *p*-value < 0.05 was considered as a significance level.

In the present study, the Bayesian approximate measurement invariance approach was applied to investigate the measurement invariance of the Q-LES-Q-SF across the ten countries. This newly introduced method, which is based on Bayesian structure equation modelling, is particularly useful when there are many groups to compare such as cross-cultural studies [21]. In traditional exact methods (e.g., multiple-group confirmatory factor analysis), researchers presume that the loadings (or intercept) are exactly equal across the groups, this means that the differences of the loadings (or intercepts) across the groups are exactly zero. Previous research showed that the exact zero constraints are very restrictive especially in many group comparisons and may leads to frequent rejection of the exact invariance model, even when the differences are ignorable across the groups. However, a promising new application of Bayesian analytic properties for assessing measurement invariance enables researchers to relax exact equality constraints. In this technique, the differences in loadings (or intercepts) of like items across the groups are allowed to be approximately zero with a mean of zero and some small variance which works through employing very narrow prior distribution to cross-group parameter differences. Because this small amount of variability is reasonably normal, a normal distribution with mean of zero and small variance is assumed for parameter differences of loadings (or intercepts).

Previous simulation studies pointed out that small variances like 0.01 or 0.05 keep differences at a minimum level; accordingly, the construct of interest remains approximately comparable across the groups [21, 22].

In this study, according to the outline of Asparouhov et al., 2015, we began running a model with a very small variance (0.001); if the model was not acceptable, we then gradually increased it to 0.01, 0.05, and 0.1 to determine the level of variation for the parameters differences of loading and intercept which would lead to acceptable model fit [23]. The fit of the Bayesian model can detect whether actual deviations are larger than those that the researcher allows in the prior distribution which is based on the posterior predictive probability (PPP) values and the confidence interval (CI) between the observed and replicated chi-square values. When zero lied in the CI and the PPP was not significant (around 0.5), the model fit well. While, when the model fit was unacceptable, the non-invariant items can be detected by identifying those loadings and intercepts which were different across the groups [21, 22]. It should be noted in contrast to traditional exact and approximate procedures in which all studied countries are compared with each other, in Bayesian approximate measurement invariance approach each country is compared with a previously set values estimated based on the mean of the posterior distribution. In other words, non-invariant items were determined as the difference of a particular parameter (loadings and/or intercept) at a specific country from the average of estimates, based on posterior distribution, for that particular parameter across the ten countries. If a difference of zero was outside of the 95% confidence interval of the posterior distribution of differences, the difference was assumed to be significant and the item can be considered to be non-invariant [24].

It should be mentioned that in the present study, the proportion of female students was significantly smaller in Indian, Nepal, and particularly Bangladesh in contrast to the other countries which should be taken into account in the assessment of measurement invariance. The importance of this issue is due to the fact that lack of invariance may be due to inherent differences in the distribution of confounding variables across the groups not due to the inherent measurement non-invariance. However, despite the appealing properties of Bayesian approximate measurement invariance approach, the effect of confounding variables cannot be controlled in the model. Therefore, we investigated the cross-cultural measurement invariance of the questionnaire in the subsample of male and female students separately in addition to the whole sample.

The Mplus 7 software was used to perform Bayesian approximate measurement invariance.

## Results

Table 1 represents the mean (± SD) age of individuals as well as percentage of males/females in each country. As shown the mean age of participant significantly differed among the countries; Brazil had the greatest mean age (25.18 ± 5.26) among all the studied countries and India had the smallest mean age (20.57 ± 2.97). In addition, the percentage of male and female were significantly different among the countries; in almost all countries the percentage of female were higher than male except for India, Nepal, and particularly for Bangladesh.

**Table 1:** Distribution of participants by gender and age across ten countries

	Age	Gender (male/female)
	Mean ± SD	N(%)
Croatia (n = 461)	21.84 ± 2.53	110(23.7)/354 (76.3)
Serbia (n = 321)	22.33 ± 2.48	92(28.7)/229 (71.3)
India (n = 487)	20.57 ± 2.97	249 (51.1)/238 (48.9)
UAE (n = 210)	20.61 ± 1.89	60 (28.6)/150 (71.4)
Nepal (n = 165)	21.94 ± 2.45	92 (55.8)/73 (44.2)
Brazil (n = 127)	25.18 ± 5.26	31 (24.4)/96 (75.6)
Bangladesh (n = 183)	21.75 ± 1.80	133 (72.7)/50 (27.3)
Turkey (n = 251)	21.55 ± 2.66	97 (38.6)/154 (61.4)
Vietnam (n = 453)	20.93 ± 2.34	152 (33.6)/301(66.4)
Poland (n = 161)	21.52 ± 2.11	43 (26.7)/118 (73.3)
	*p*-value < 0.001^*^	*p*-value < 0.001^**^

* *p*-value based on one-way ANOVA (F-value = 43.07, df = 9)

** *p*-value based on chi-square test (Chi-square value = 234.56, df = 9)

Table 2 presents the value of fit indices including PPP and 95%CI for assessing Bayesian approximate measurement invariance for various magnitudes of the prior variance in the whole sample and the subsamples of male and female students. For all values of the variance of prior distribution from the smallest prior variance (0.001) to the largest value (0.1), the PPP values were zero and the confidence interval did not include zero. This suggested that approximate scalar measurement invariance did not hold for all values of variance.

**Table 2:** Fit indices of Bayesian approximate measurement invariance

		Prior	PPP	95% CI
Whole sample	Scalar invariance	N(0, 0.001)	< 0.001	(2423.02, 2665.045)
	Scalar invariance	N(0, 0.01)	< 0.001	(1876.118, 2162.83)
	Scalar invariance	N(0, 0.05)	< 0.001	(1794.11, 2041.799)
	Scalar invariance	N(0, 0.1)	< 0.001	(1787.624, 2038.699)
Male subsample	Scalar invariance	N(0, 0.001)	< 0.001	(1252.885, 1467.639)
	Scalar invariance	N(0, 0.01)	< 0.001	(1030.901, 1270.190)
	Scalar invariance	N(0, 0.05)	< 0.001	(944.417, 1174.272)
	Scalar invariance	N(0, 0.1)	< 0.001	(933.837, 1170.367)
Female subsample	Scalar invariance	N(0, 0.001)	< 0.001	(1729.296, 2017.262)
	Scalar invariance	N(0, 0.01)	< 0.001	(1307.140, 1538.557)
	Scalar invariance	N(0, 0.05)	< 0.001	(1180.035, 1416.096)
	Scalar invariance	N(0, 0.1)	< 0.001	(1172.120, 1402.634)

*PPP:* posterior predictive probability, *CI:* confidence interval

Table 3 shows all deviation of intercepts and factor loadings from the defined priors (mean = 0 variance = 0.1) in Bayesian approximate measurement invariance approach for the whole sample. Non-invariant items across the ten countries were shown with asterisk. In addition, significant deviation of the factor loadings and/or intercepts from the average were shown by × in various countries. As shown only items 4 and 7 were invariant across all countries; while, the other items were non-invariant at least in one country. This means that at least in one country the estimated posterior parameters of interest (factor loading and/or intercept) were deviated substantially from the average posterior estimates across all the countries and these items were non-invariant. For instance, the intercept of item 6 (family relationship) deviated from the defined prior only in Bangladesh which means that this item was interpreted differently by the university students in this country as opposed to the other nine countries. On the other side, the parameters of item 12 (ability to get around physically without feeling dizzy or unsteady or falling) deviated from the defined prior in almost all countries, except for Nepal and Vietnam. This suggested that this item was perceived in a different way in almost all countries. Item 1 in India and Turkey, item 3 (work) in Vietnam and Poland, and item 5 (social relationships) in Indian and Poland were not invariant. In addition, items 9 (sexual drive, interest and/or performance) and 11 (living/housing situation) were non-invariant in Bangladesh, Vietnam, and Poland. The parameters of item 2 (mood) in India, Bangladesh, Vietnam, and Poland along with item 14 (overall sense of well-being) in India, Bangladesh, and Poland deviated from the defined prior. Furthermore, item 10 (economic status) was perceived differently in Croatia, Serbia, India, UAE, and Vietnam. Item 13 (your vision in terms of ability to do work or hobbies) were also perceived in a different way in Serbia, India, Turkey, and Vietnam. Poland was the country in which the greatest number of non-invariant items were detected following by India and Bangladesh. It should be noted that almost the same non-invariant items were detected for the other values of prior variance including, 0.001, 0.01, and 0.05 which were not shown here.

**Table 3:** Deviation of factor loadings and intercepts from prior defined parameters (mean = 0, variance = 0.1) in the whole sample

	Croatia		Serbia		India		UAE		Nepal		Brazil		Bangladesh		Turkey		Vietnam		Poland	
	lo	int	lo	int	lo	int	lo	int	lo	int	lo	int	lo	int	lo	int	lo	int	lo	int
q_1_^*^						** × **									** × **					
q_2_^*^						** × **								** × **				** × **		** × **
q_3_^*^																		** × **		** × **
q_4_																				
q_5_^*^						** × **														** × **
q_6_^*^														** × **						
q_7_																				
q_8_^*^		** × **																		** × **
q_9_^*^														** × **				** × **		** × **
q_10_^*^		** × **		** × **		** × **		** × **									** × **			
q_11_^*^														** × **				** × **		** × **
q_12_^*^		** × **	** × **	** × **	** × **	** × **	** × **	** × **			** × **	** × **	** × **	** × **		** × **				** × **
q_13_^*^				** × **		** × **									** × **	** × **		** × **		
q_14_^*^					** × **	** × **							** × **							** × **

*lo*: factor loading, *int*: intercept, × : deviation of a given parameter in a given country from the defined priors (mean = 0, variance = 0.1)

Tables 4 and 5 present the results of Bayesian approximate measurement invariance technique for the subsample of male and female students, respectively (mean = 0 variance = 0.1). Non-invariant items across the ten countries were shown with asterisk. In addition, significant deviation of the factor loadings and/or intercepts from the average were shown by × in various countries. An interesting finding is that fewer non-invariant items were detected in both subsamples (particularly in the subsample of male students) as compared with the results of whole sample. As shown in Table 4 items 4, 5, 10, 13, and 14 were invariant across all countries and the other items were non-invariant at least in one country. In contrast, only items 4, 7, and 14 were invariant among subsample of female students (Table 5); while the other items were detected as non-invariant across the countries.

**Table 4:** Deviation of factor loadings and intercepts from prior defined parameters (mean = 0, variance = 0.1) in the male subsample

	Croatia		Serbia		India		UAE		Nepal		Brazil		Bangladesh		Turkey		Vietnam		Poland	
	lo	int	lo	int	lo	int	lo	int	lo	int	lo	int	lo	int	lo	int	lo	int	lo	int
q_1_^*^															×		×			
q_2_^*^																				×
q_3_^*^																		×		
q_4_																				
q_5_																				
q_6_^*^														×						×
q_7_^*^														×						
q_8_^*^																				×
q_9_^*^							×			×				×		×	×			
q_10_																				
q_11_^*^								×												
q_12_^*^		×		×	×	×		×						×	×		×			×
q_13_																				
q_14_																				

*lo*: factor loading, *int:* intercept, × : deviation of a given parameter in a given country from the defined priors (mean = 0, variance = 0.1)

**Table 5:** Deviation of factor loadings and intercepts from prior defined parameters (mean = 0, variance = 0.1) in the female subsample

	Croatia		Serbia		India		UAE		Nepal		Brazil		Bangladesh		Turkey		Vietnam		Poland	
	lo	int	lo	int	lo	int	lo	int	lo	int	lo	int	lo	int	lo	int	lo	int	lo	int
q_1_^*^						×										×				×
q_2_^*^						×												×		
q_3_^*^																			×	
q_4_																				
q_5_^*^																×				×
q_6_^*^																		×	×	
q_7_																				
q_8_^*^		×								×										
q_9_^*^	×	×				×						×		×			×	×		×
q_10_^*^		×		×		×		×				×		×				×		×
q_11_^*^		×					×											×		×
q_12_^*^		×		×	×	×		×				×		×		×				×
q_13_^*^				×		×										×		×		
q_14_																				

*lo:* factor loading, *int:* intercept, × : deviation of a given parameter in a given country from the defined priors (mean = 0, variance = 0.1)

## Discussion

This study is the first to investigate the measurement invariance of the Q-LES-Q-SF across several countries with diverse socioeconomic, cultural, and religious backgrounds. Our findings revealed that 12 out of 14 items (85%) were non-invariant across the studied countries, what implies that individuals from different countries likely have different perceptions of the items. Thus, the Q-LES-Q-SF is likely not an invariant measure for cross-cultural QOL comparisons.

Only item 4 (household activities) and item 7 (leisure time activities) were invariant across the countries. Although there are no clear data regarding the reason for this finding, one possible explanation could be that irrespective of culture or socioeconomic status of countries, individuals from different countries had the same feeling about levels of satisfaction with doing household and leisure time activities. On the other hand, item 12 (ability to get around physically without feeling dizzy or unsteady or falling) was non-invariant across almost all the countries, except Nepal and Vietnam. Different interpretations of this items in different countries may be attributed to variation in factors such as the perception of health, description of symptoms, or cultural schemata which is apparent in folk illnesses [5]. The remaining items were also non-invariant at least in one country. This discrepancy in the performance of the items across different countries could be attributed to a wide variety of factors. Previous research showed that diverse contextual characteristics of different countries such as cultural, historical, religious, and socio-economic variables along with different values placed on QOL may lead to different individual perceptions of the items in QOL measures [5, 7, 25–27]. Translation inequivalence including both wording of an item or the response category labels might be another reason for item bias across countries with different languages [28]. Although previous studies showed that even the best possible translation may not be accurately equivalent to the original version of the questionnaire, the more similar the language of respondents was to the original language in which the questionnaire was developed, the less non-invariant items were observed [28–30]. For instance, Scott et al. reported that the most non-invariant items in the European Organization for Research and Treatment of Cancer Core Quality of Life questionnaire (EORTC QLQ-C30) were detected between Eastern European and Asian countries [30]. Furthermore, different response styles in terms of social desirability responding, acquiesces response set (the tendency to agree with questions) and extreme response bias in different culture can be potential sources of measurement non-invariance of a given questionnaire. Generally, it has been shown that Asian respondents are more likely to agree with the question and less likely to select extreme values in rating scale [31]. This could be a possible explanation for the findings of this study showing that in Nepal, the factor loadings and intercepts of all the items did not deviate from the defined priors (mean = 0 and variance = 0.1).

Another important finding is that fewer non-invariant items were detected when cross-cultural measurement invariance testing was performed on the subsample of male and female students separately (especially among subsample of male students). Although this result may be due to the fact that lack of invariance across the studied countries can be attributed to the differences in the interpretation of the items between male and female students, small and unbalanced sample size may also negatively influence the power of the test for detecting non-invariant items.

It should be mentioned that there was no similar study on the cross-cultural invariance of the Q-LES-Q-SF questionnaire for comparison. However, several studies have examined the measurement invariance of the other QOL questionnaires such as the World Health Organization Quality-of-Life Scale (WHOQOL), KINDL, KIDSCREEN, EORTC QLQ-C30, Pediatric Quality of Life Inventory (PedsQL), and EUROHIS-QOL across various cultural, language, and ethnic groups [5, 25–27, 32–39]. Yet, inconsistent and contradictory findings have been offered by these studies. An example is a study conducted by Gibbons et al. in which the measurement invariance of the WHOQOL was assessed across four diverse countries including UK, Zimbabwe, Russia, and India. They concluded that the majority of the items (75%) were non-invariant in the studied countries [32]. Moreover, Benítez-Borrego et al. reported that the WHOQOL-BREF was not invariant across nine Spanish-speaking countries in spite of their common language [25]. Scott et al. also conducted two different studies for investigating the measurement invariance of the EORTC QLQ-C30, which is one of the most widely used QOL questionnaires cancer patients, across more than 20 countries all over the world [28, 30]. Their findings revealed that the results of the United Kingdome (UK), North America, and Australia were fairly similar which may be due to their common language and culture. While, more non-invariant items were detected for Eastern European countries as opposed to Western European ones. In addition, the most number of non-invariant items were found across Eastern European and Asian countries [28, 30]. Furthermore, in research which examined the measurement invariance of the EUROHIS-QOL 8-item questionnaire across ten European countries moderate non-invariance was detected. It has been shown that when the culture was closer to the western draft language culture, less non-invariant items were identified [29]. Stevanovic et al. also reported that the PedsQL, as one of the most frequently used pediatric QOL questionnaire, was not invariant across seven countries [27]. Similar results were found for the measurement invariance of the KINDL questionnaire across Serbian and Iranian children and adolescents [26]. In contrast, there are a number of studies supporting the cross-cultural invariance of some QOL measure. For example, it has been shown that WHOQOL-AGE used for measuring QOL in aging population was partially invariant across three countries, namely, Finland, Poland, and Spain [36]. The KIDSCREEN questionnaire introduced for evaluating children and adolescent QOL is another example of cross-cultural invariant measure. Two independent studies have indicated that this questionnaire was invariant across Serbian and Iranian children and adolescents [37] as well as thirteen European countries [34]. These contradictory results could be attributed to various possible explanations such as various QOL questionnaires applied in the mentioned studies, differences in health conditions and age of respondents along with different statistical methods used to assess measurement invariance of the questionnaires.

It should be noted that the key strength of the current study is utilizing a large sample size which included socioeconomically, culturally, and religiously diverse nations. These components enhance the generalizability of our findings to a multicultural context. Furthermore, the main advantage of the Bayesian approximate measurement invariance used in this study is that researchers are allowed to relax exact equality constraint by presuming that factor loadings and intercept are only approximately equal; yet, differences are still maintained at a minimum to make sure that the underlying construct remains approximately comparable across the groups.

Several limitations to this study need to be acknowledged. First, the studied countries differed significantly in terms of age and gender of the participants. The importance of this issue is due to the fact that without controlling the effects of covariates, the results of measurement invariance can be distorted [40]. Second, the socioeconomic status of the participants was not collected which may be a possible explanation for lack of invariance; therefore, it is highly recommended that in future studies socioeconomic status of participants be evaluated while testing measurement invariance. Third, the measurement invariance of the Q-LES-Q-SF was not evaluated across different countries with the same language or across different languages in the same region. Hence, it is onerous to realize that different interpretations of the items were due to a lack of translation equivalence or cultural diversity. Accordingly, conducting structured interviews with bilingual people in future studies could be fruitful to answer this question. Moreover, since the results of measurement invariance testing could vary substantially from one questionnaire to another; therefore, further investigations should be conducted to assess the cross-cultural measurement invariance of the other QOL questionnaires. Finally, sample size in different countries was unbalanced, especially in the subsample of male and female students, which may adversely affect the power of the measurement invariance testing for detecting lack of invariance. Although previous simulation research showed that unbalanced sample size led to substantial reduction in the power of measurement invariance testing across two groups [41], no study has been carried out to investigate this issue when the number of groups being tested is large. Hence, it would be interesting to investigate the effects of unbalanced sample size on the power of Bayesian approximate measurement invariance approach in future studies.

## Conclusion

Our findings did not support the cross-cultural measurement invariance of the Q-LES-Q-SF, thus considerable caution is warranted when comparing QOL across different countries with this measure. It is suggested that before any comparisons, the cross-cultural measurement invariance is tested first and the questionnaire is calibrated for non-invariant items. The results of this study may help in future research with Q-LES-Q-SF in item rewording and adaptation as well as calibrating non-invariant items to narrow the differences and help create an invariant measure for valid QOL comparisons across different countries since using culture-specific measures is not practical for cross-cultural studies.

## Data Availability

The datasets generated and/or analyzed during the current study are not publicly available due to sensitive information included as related to some medical and personal data of the participants, but are available from the corresponding author on reasonable request.

## Abbreviations

QOL: Quality of Life
WHO: World Health Organization
Q-LES-Q-SF: Quality of Life Enjoyment and Satisfaction Questionnaire-Short Form
PPP: Posterior predictive probability
CI: Confidence interval
EORTC QLQ-C30: European Organization for Research and Treatment of Cancer Core Quality of Life questionnaire
WHOQOL: World Health Organization Quality-of-Life Scale
PedsQL: Pediatric quality of life inventor

## References

[1] Haraldstad K, Wahl A, Andenæs R, Andersen JR, Andersen MH, Beisland E (2019). A systematic review of quality of life research in medicine and health sciences. Qual Life Res.

[2] Demyttenaere K, Andersen HF, Reines EH (2008). Impact of escitalopram treatment on quality of life enjoyment and satisfaction questionnaire scores in major depressive disorder and generalized anxiety disorder. Int Clin Psychopharmacol.

[3] The World Health Organization Quality of Life assessment (WHOQOL): position paper from the World Health Organization. Soc Sci Med. 1995;41:1403–1409. 10.1016/0277-9536(95)00112-k 8560308

[4] Beaton DE, Bombardier C, Guillemin F, Ferraz MB (2000). Guidelines for the process of cross-cultural adaptation of self-report measures. Spine.

[5] Schmidt S, Bullinger M (2003). Current issues in cross-cultural quality of life instrument development. Arch Phys Med Rehabil.

[6] Ho SMY, Rochelle TL, Law LSC, Wenjie Duan Y, Bai S-MS, Pedrotti JT, Edwards LM (2014). Methodological issues in positive psychology research with diverse populations: exploring strengths among Chinese adults. Perspectives on the intersection of multiculturalism and positive psychology.

[7] Teresi JA, Fleishman JA (2007). Differential item functioning and health assessment. Qual Life Res.

[8] Endicott J, Nee J, Harrison W, Blumenthal R (1993). Quality of life enjoyment and satisfaction questionnaire: a new measure. Psychopharmacol Bull.

[9] Stevanovic D (2011). Quality of life enjoyment and satisfaction questionnaire-short form for quality of life assessments in clinical practice: a psychometric study. J Psychiatr Ment Health Nurs.

[10] Lee YT, Liu SI, Huang HC, Sun FJ, Huang CR, Yeung A (2014). Validity and reliability of the Chinese version of the short form of quality of life enjoyment and satisfaction questionnaire (Q-LES-Q-SF). Qual Life Res.

[11] Bourion-Bédès S, Schwan R, Epstein J, Laprevote V, Bédès A, Bonnet JL (2015). Combination of classical test theory (CTT) and item response theory (IRT) analysis to study the psychometric properties of the French version of the quality of life enjoyment and satisfaction questionnaire-short form (Q-LES-Q-SF). Qual Life Res.

[12] Bourion-Bédès S, Schwan R, Laprevote V, Bédès A, Bonnet JL, Baumann C (2015). Differential item functioning (DIF) of SF-12 and Q-LES-Q-SF items among french substance users. Health Qual Life Outcomes.

[13] Petrović A, Janković S (2017). Translation, cultural adjustment and evaluation of reliability and validity of “quality of life enjoyment and satisfaction questionnaire – short form” for patients with schizophrenia. Acta Fac Medicae Naissensis.

[14] Riendeau RP, Sullivan JL, Meterko M, Stolzmann K, Williamson AK, Miller CJ (2018). Factor structure of the Q-LES-Q short form in an enrolled mental health clinic population. Qual Life Res.

[15] Rush AJ, South CC, Jha MK, Grannemann BD, Trivedi MH (2019). Toward a very brief quality of life enjoyment and satisfaction questionnaire. J Affect Disord.

[16] Zubaran C, Foresti K, Thorell MR, Franceschini PR, Homero W (2009). Portuguese version of the quality of life enjoyment and satisfaction questionnaire: a validation study. Rev Panam Salud Publica.

[17] Mick E, Faraone SV, Spencer T, Zhang HF, Biederman J (2008). Assessing the validity of the quality of life enjoyment and satisfaction questionnaire short form in adults with ADHD. J Atten Disord.

[18] Balhara YPS, Doric A, Stevanovic D, Knez R, Singh S, Chowdhury MRR (2019). Correlates of problematic internet use among college and university students in eight countries: an international cross-sectional study. Asian J Psychiatr.

[19] Stevanović D, Đoric A, Balhara YPS, Ćirović N, Arya S, Ransing R (2020). Assessing the symptoms of internet gaming disorder among college/university students: an international validation study of a self-report. Psihologija.

[20] Winter SD, Depaoli S (2019). An illustration of Bayesian approximate measurement invariance with longitudinal data and a small sample size. Int J Behav Dev.

[21] Muthén B, Asparouhov T. BSEM measurement invariance analysis. Mplus web notes: No.17. 2013. Available on line at: www.statmodel.com

[22] van de Schoot R, Kluytmans A, Tummers L, Lugtig P, Hox J, Muthen B (2013). Facing off with Scylla and Charybdis: a comparison of scalar, partial, and the novel possibility of approximate measurement invariance. Front Psychol.

[23] Asparouhov T, Muthén B, Morin AJS (2015). Bayesian structural equation modeling with crossloadings and residual covariance comments on Stromeye et al. J Manag.

[24] Seddig D, Leitgö H (2018). Approximate measurement invariance and longitudinal confirmatory factor analysis: concept and application with panel data. Surv Res Methods.

[25] Benítez-Borrego S, Mancho-Fora N, Farràs-Permanyer L, Urzúa-Morales A, Guàrdia-Olmos J (2016). Differential item functioning of WHOQOL-BREF in nine Iberoamerican countries. Rev Iberoam Psicol Salud.

[26] Jafari P, Stevanović D, Bagheri Z (2016). Cross-cultural measurement equivalence of the KINDL questionnaire for quality of life assessment in children and adolescents. Child Psychiatry Hum Dev.

[27] Stevanovic D, Atilola O, Vostanis P, Balhara YPS, Avicenna M, Kandemir H, Knez R, Franic T, Petrov P, Maroco J, Supic ZT, Bagheri Z (2016). Cross-cultural measurement invariance of adolescent self-report on the pediatric quality of life inventory™ 4.0. J Res Adolesc.

[28] Scott NW, Fayers PM, Bottomley A, Aaronson NK, de Graeff A, Groenvold M (2006). Comparing translations of the EORTC QLQ-C30 using differential item functioning analyses. Qual Life Res.

[29] Schmidt S, Mühlan H, Power M (2006). The EUROHIS-QOL 8-item index: psychometric results of a cross-cultural field study. Eur J Public Health.

[30] Scott NW, Fayers PM, Aaronson NK, Bottomley A, de Graeff A, Groenvold M, Koller M, Petersen MA, Sprangers MAG (2007). The use of differential item functioning analyses to identify cultural differences in responses to the EORTC QLQ-C30. Qual Life Res.

[31] Azocar F, Areán P, Miranda J, Muñoz RF (2001). Differential item functioning in a Spanish translation of the Beck Depression Inventory. J Clin Psychol.

[32] Gibbons CJ, Skevington SM (2018). Adjusting for cross-cultural differences in computer-adaptive tests of quality of life. Qual Life Res.

[33] Newman DA, Limbers CA, Varni JW (2010). Factorial invariance of child self-report across English and Spanish language groups in a Hispanic population utilizing the PedsQL™ 4.0 generic core scales. Eur J Psychol Assess.

[34] Ravens-Sieberer U, Auquier P, Erhart M, Gosch A, Rajmil L, Bruil J (2007). The KIDSCREEN-27 quality of life measure for children and adolescents: psychometric results from a cross-cultural survey in 13 European countries. Qual Life Res.

[35] Ofenloch RF, Oosterhaven JAF, Svensson A, Weisshaar E, Minamoto K (2017). Cross-cultural validation of the quality of life in hand eczema questionnaire (QOLHEQ). J Invest Dermatol.

[36] Santos D, Abad FJ, Miret M, Chatterji S, Olaya B, Zawisza K (2018). Measurement invariance of the WHOQOL-AGE questionnaire across three European countries. Qual Life Res.

[37] Stevanovic D, Jafari P (2015). A cross-cultural study to assess measurement invariance of the KIDSCREEN-27 questionnaire across Serbian and Iranian children and adolescents. Qual Life Res.

[38] Tripathy S, Myatra SN (2020). Are the instruments for quality of life assessment comparable between cultures?. No Intensive Care Med.

[39] Watt T, Barbesino G, Bjorner JB, Bonnema SJ, Bukvic B, Drummond R (2015). Cross-cultural validity of the thyroid-specific quality-of-life patient-reported outcome measure. ThyPRO Qual Life Res.

[40] Huang IC, Leite WL, Shearer P, Seid M, Revicki DA, Shenkman EA (2011). Differential item functioning in quality of life measure between children with and without special health-care needs. Value Health.

[41] Yoon M, Lai MH (2018). Testing factorial invariance with unbalanced samples. Struct Equ Model.

